# Grieving Process During the COVID-19 Pandemic: Development and Preliminary Findings of a Group Intervention Based on Cognitive-Narrative Theory

**DOI:** 10.3390/bs16040516

**Published:** 2026-03-29

**Authors:** Inês Marques, Cristina A. Godinho, Rita Francisco

**Affiliations:** 1 Faculty of Human Sciences, Universidade Católica Portuguesa, 1649-023 Lisboa, Portugal; imarques@ulslo.min-saude.pt; 2 Lisbon West Local Health Unit, Hospital São Francisco Xavier, 1449-005 Lisboa, Portugal; 3 National School of Public Health, Public Health Research Centre, CISP, Comprehensive Health Research Center, CHRC, NOVA University Lisbon, 1600-560 Lisboa, Portugal; cristina.godinho@ensp.unl.pt

**Keywords:** bereavement, prolonged grief disorder, coping strategies, COVID-19 pandemic, cognitive-narrative intervention, group intervention, reconstruction of meaning

## Abstract

The COVID-19 pandemic has been associated with a substantial number of deaths, exposing many individuals to bereavement under particularly adverse circumstances, as public health restrictions often prevented individuals from engaging in customary farewell and mourning practices. In this context, the development of interventions capable of mitigating the psychological impact of grief is of critical importance. This mixed-methods study, with a predominantly qualitative design, aimed to develop and pilot-test a group intervention grounded in cognitive-narrative theory for individuals experiencing bereavement during the COVID-19 pandemic, in Portugal. Four patients aged between 18 and 65 years (M = 49.25; SD = 21.24) participated in the 6-week intervention, between July and August 2022. Quantitative data were collected using the Grief and Meaning Reconstruction Inventory, the Prolonged Grief Assessment Instrument, and the Hospital Anxiety and Depression Scale, with pre- and post-intervention comparisons. To assess the intervention process, participants completed an individual evaluation form, and a group interview was conducted at the end of the intervention. The results indicated a clinically significant reduction in feelings of emptiness and loss of meaning in most participants, with improved meaning-making related to the loss. The thematic analysis performed on the qualitative data highlighted the strengths of the intervention (e.g., adjustment to grief and sharing) and some areas for improvement (e.g., more regular feedback and group composition). Despite limitations, particularly the small sample size, the findings are promising and support further evaluation of this intervention in larger samples of individuals diagnosed with prolonged grief.

## 1. Introduction

Despite being a natural event, the death of a family member or close friend is, of all human experiences, one of the main challenges of adaptation and one of the most painful in life (Chen, 2022; Walsh, 2020). Bereavement encompasses the experience itself, the expressions of bereavement, and the internal psychological and adaptive processes (Christ et al., 2003). The complications resulting from bereavement exist on a continuum, which can progress from mild and transient to severe and disabling (Neimeyer, 2019), with the diagnosis of prolonged grief disorder being the most extreme point on this continuum.

The COVID-19 pandemic had an overwhelming impact on all regions of the world between 2020 and 2022. As a result of the increase in mortality rates worldwide, the number of bereaved individuals has substantially increased (Stroebe & Schut, 2021). In Portugal, by the end of 2022, 25.796 deaths associated with COVID-19 had been recorded (National Institute of Statistics, 2023). Considering estimates that each death affects approximately six bereaved individuals (Barry et al., 2002), it can be inferred that during this period approximately 154,776 individuals experienced the loss of a loved one. This figure becomes even more concerning when deaths attributable to other causes are considered. Moreover, the pandemic meant that some restrictions had to be implemented, such as the absence of funeral rituals, implying a reduction in the social support received, as well as various difficulties in terms of emotional expression, acceptance of the loss, and its integration into the identity of the bereaved (Gabriel & Paulino, 2021). These circumstances tended to exacerbate negative emotional experiences, including feelings of guilt, anger, helplessness, emptiness, and indignation. In Portugal, funeral rituals were entirely suspended between March and May 2020 due to public health restrictions that required the closure of churches. Following this period and extending until January 2023, funeral ceremonies were gradually resumed; however, they were conducted under strict restrictions. These measures included the mandatory closure of coffins, the maintenance of a physical distance of at least 1.5 m between mourners, and the avoidance of large gatherings (Directorate General for Health, 2022).

These circumstances significantly affected how individuals and families experienced loss and bereavement, often disrupting traditional mourning practices and limiting opportunities for social support. In response to these challenges, some psychosocial interventions were developed to support individuals bereaved during the pandemic. For example, Lordello and Silva (2021) described a narrative-based group intervention, carried out virtually in Brazil, aimed at helping participants share their loss experiences and reconstruct meaning following the death of a loved one during the COVID-19 pandemic. The present study was conducted within this same context and aims to contribute to the emerging literature on psychological interventions designed to support individuals whose grieving processes were shaped by the constraints associated with the COVID-19 pandemic.

Given the new reality that has taken hold for more than three years, it is reasonable to assume that all these changes have exposed the bereaved to more risk factors for the emergence of a complicated grieving process (Stroebe & Schut, 2021; Walsh, 2020): (1) the absence of funeral rituals considered relevant to most individuals, which potentially generated anxiety, due to the constraints at the time of farewell, hindering the opportunity for emotional expression and receiving social and emotional support (Gabriel & Paulino, 2021); (2) the inability to attribute meaning to the loss, since the uncertainty associated with the pandemic triggered in individuals a need to manage the anxiety felt on a daily basis (Gabriel & Paulino, 2021), contributing to an emotional and cognitive unavailability for the task of assigning meaning; (3) the presence of feelings of guilt and anger that tend to be present in situations of suspected contagion, or in situations where the individual feels they could have saved the deceased (Taylor, 2017); (4) reduced social support due to the fact that many individuals have gone through a grieving process during the pandemic, and the community is not emotionally available to support others; and (5) resorting to isolation within one’s own home, since voluntary isolation after a loss is not the same as isolation imposed as a public health measure to control a pandemic, and feelings of pain and loneliness should not be devalued (Gabriel & Paulino, 2021).

The ability to minimize the negative emotions felt during the grieving process appears to have some benefits, particularly in terms of the ease with which the bereaved individual can maintain functioning, and the ability to adapt to the concrete adversities arising from the loss of a loved one by adapting to family changes (Pastor-Oliva et al., 2025). Thus, given the uniqueness of the grieving process in times of the pandemic, the literature lists some strategies used by individuals that proved to be fundamental in the grieving process. One of the strategies adopted by families was the search for alternative rituals to make funeral ceremonies less lonely, allowing individuals to say goodbye to the deceased (e.g., passing by the deceased’s family home on the way to the crematorium), with the aim of relieving the pain felt (Borghi & Menichetti, 2021). Exploring spirituality also allows families to acquire a set of resources to understand and deal with their losses (Park & Halifax, 2011). By using this type of resource, the bereaved find comfort and consolation, which, over time, allows them to accept and face death more peacefully (Park & Halifax, 2011). Research suggests that developing a positive meaning about a loss can lead to a greater sense of well-being, greater involvement with others and a greater sense of purpose (Calhoun et al., 2010). In fact, some bereaved people engage in meaning-making activities to convey meaning to their own existence (Gillies & Neimeyer, 2006).

Given the clinical relevance of grief and its significance in terms of emotional suffering, it is essential to explore and develop appropriate therapeutic interventions for prolonged grief (Boelen & Lenferink, 2020). Cognitive-narrative intervention helps to identify a meaning for the loss itself, through the exploration of emotions and thoughts (Neimeyer, 2019; Rocha & Gabriel, 2021). This approach allows the individual to master complicated content about the loss and enables them not to avoid facing their pain (Neimeyer et al., 2010), providing better emotional regulation and less rumination associated with the event (Gonçalves et al., 2000). The intervention developed in this study was based on two models: (1) the cognitive-narrative bereavement intervention model developed by Barbosa et al. (2014), which in turn was based on the constructivist model of Gonçalves (1997); and (2) the reconstruction of meaning model developed by Neimeyer and Thompson (2014). The individual cognitive-narrative bereavement intervention model includes four phases, namely, recollection, emotional and cognitive subjectivation, metaphorization and projection, and is expected to last four sessions. In addition, an attempt was made to strengthen the structure of the intervention in terms of the meaning model (Neimeyer & Thompson, 2014), by adding two essential components to bereavement therapy, which were interconnected with the phases of the cognitive-narrative bereavement intervention model. These components are the processing of the story of the loss and the story of the relationship with the deceased, allowing for the restoration of a sense of attachment to the deceased. The intervention proposed in this study also included activities using expressive arts and therapeutic letters (White & Epston, 1990), with the aim of making it easier for patients to understand their evolution throughout the intervention and the preponderant role they play in it.

Although several psychological interventions have been developed to support bereaved individuals, studies examining interventions specifically designed for grief experienced during the COVID-19 pandemic remain limited. In addition, cognitive-narrative approaches to bereavement intervention have mainly been implemented in individual therapy formats, with fewer studies exploring their application in group settings. The present study seeks to address this gap by developing and preliminarily evaluating a group intervention grounded in cognitive-narrative theory, adapted for individuals who experienced the loss of a loved one during the COVID-19 pandemic. Specifically, we intended to: (a) design the intervention; (b) evaluate its potential impact on reducing the anxious and depressive symptoms associated with the grieving process and prolonged bereavement, as well as on creating a system of meanings for the loss; and (c) identify the strengths of the intervention and aspects for improvement from the patients’ perspective.

## 2. Materials and Methods

### 2.1. Intervention Proposal

The cognitive-narrative bereavement intervention model was originally developed for individual therapy (Neimeyer, 2019; Rocha & Gabriel, 2021); therefore, adaptations were implemented to enable its application within a therapeutic group setting. The intervention was structured into six sessions, lasting 90 min (Table 1), and was led by the first author. Apart from the last session, all the sessions included between-session assignments designed to consolidate what they had worked on during the previous sessions and to prepare for the next session. Another benefit of these activities was that the patients were left with materials and tools that could be used in other moments of vulnerability.

### 2.2. Procedure

After the study was approved by the Ethics Committee of the Hospital Center where the intervention was carried out (protocol code 3.2022), participants were recruited through referrals from psychiatrists, between May and June 2022. Informed consent was requested at the screening appointment prior to the start of the intervention. The group intervention took place at the hospital between July and August 2022 and consisted of six weekly sessions, each lasting approximately 90 min.

### 2.3. Participants

Inclusion criteria were defined as patients attending the Psychiatry consultation at the aforementioned Hospital Center, aged 18 or over, and who had experienced the death of a significant person during the COVID-19 pandemic. The exclusion criteria were not being able to speak Portuguese, having impaired comprehension, having a diagnosis of psychosis, the presence of dementia, or problems with substance abuse or dependence. Initially, each attending psychiatrist was asked to refer patients with a possible diagnosis of pathological bereavement to the psychology team. These patients subsequently underwent a screening process to identify those who were more likely to benefit from participation in a group-based intervention rather than individual follow-up. Six patients met the inclusion criteria and were initially invited to participate in the intervention. However, one participant was unable to attend due to work schedule constraints, and another preferred to receive individual psychological support rather than participate in the group intervention. Consequently, the final sample consisted of four participants.

Three women and one man, aged between 18 and 65 (M = 49.25, SD = 21.24), took part in the group intervention. Table 2 shows the participants’ sociodemographic data, as well as other data relating to the situation of loss.

### 2.4. Instruments

#### 2.4.1. General Questionnaire

Initially, participants were asked to answer a general questionnaire, which allowed for the collection of the patient’s sociodemographic data (age, gender, marital status, level of education and nationality) and data contextualizing the individual’s loss (e.g., type of relationship with the significant person who died, how long ago the death occurred, whether the bereaved individual received psychological help, circumstance/cause of death).

#### 2.4.2. Grief and Meaning Reconstruction Inventory (GMRI)

Developed by Gillies et al. (2015), the GMRI aims to assess the attribution of meaning through a set of 29 items with positively framed statements (e.g., “Since the loss, I am a strong person.”) and negatively framed statements (e.g., “I cannot understand this loss.”), to which participants must respond taking into account their experiences in the last week, using Likert-type scales from 1 (“strongly disagree”) to 5 (“strongly agree”). The instrument has five subscales, namely continuing bonds, personal growth, sense of peace, emptiness and meaninglessness, and valuing life. According to the authors of the original scale, both the overall GMRI (α = 0.84) and its constituent factors (from α = 0.76 to α = 0.85) showed good internal consistency (Gillies et al., 2015). The GMRI was translated into Portuguese by one member of the research team who is fluent in both English and Portuguese. The translated version was subsequently reviewed by two experts with experience in the relevant research area to evaluate semantic and conceptual equivalence with the original English instrument. Minor wording adjustments were made based on their feedback to ensure clarity and consistency with the meaning of the original items.

#### 2.4.3. Prolonged Grief Scale—Revised (PG13-R)

The PG13-R is a diagnostic tool for prolonged grief, made up of 13 items and divided into three parts (Prigerson et al., 2007). The first part is made up of four items (e.g., “In the last month, how often have you missed and felt the absence of the person you lost?”), assessed using a Likert scale from 1 (“almost never”) to 5 (“several times a day”), allowing to assess the frequency of the feeling of separation anxiety, and one item assessed using a dichotomous yes/no answer, referring to the duration of this feeling. The second part is made up of six items describing the cognitive, emotional and behavioral symptoms of bereavement (e.g., “Do you feel bitter about your loss?”), assessed on a Likert scale ranging from 1 (“not at all”) to 5 (“extremely”). The last part consists of an item with a dichotomous answer (yes/no) referring to functional incapacity in the social, occupational or other areas of functioning (e.g., “Have you felt a significant reduction in your social, professional or other important areas of life?”). The internal consistency of PG13-R in the Portuguese version, used in the present study, is high (α = 0.93; Delalibera et al., 2011).

#### 2.4.4. Hospital Anxiety and Depression Scale (HADS)

The HADS (Zigmond & Snaith, 1983) aims to assess changes in the patient’s emotional state over the last week, through 14 items, divided into two subscales: anxiety (e.g., “I feel tense or nervous”) and depression (e.g., “I still enjoy the things I used to enjoy”), to which patients respond on a Likert scale between 0 (“almost never”) and 3 (“almost always”). The overall score for both subscales varies between 0 and 21, and the cut-off point for a clinical level of anxiety or depression is 11. The Portuguese version of HADS, used in the present study, presents adequate levels of internal reliability (α = 0.76 anxiety subscale; α = 0.81 depression subscale; Pais-Ribeiro et al., 2007).

#### 2.4.5. Individual Final Evaluation Form

This evaluation form was created as part of this study and includes open-ended questions to assess each patient’s perception of the intervention and the progress made, the most positive and negative aspects of the intervention, as well as the adjustments needed to improve (e.g., “What were the most positive aspects of the intervention?”, “What were the most negative aspects?”, “What aspects should be improved?”, “Would you recommend this therapeutic group to someone experiencing bereavement? Why?”). Additional questions explored participants’ views on the adequacy of the duration of each session, the overall duration of the intervention, and the usefulness of the between-session assignments. Participants completed the form individually and provided written responses at the end of the intervention.

#### 2.4.6. Final Group Evaluation Interview Script

The purpose of the final evaluation interview was to expand on the answers given in the individual final evaluation form. A semi-structured group interview script was developed, covering topics such as the advantages of joining the therapeutic group, points for improvement in the intervention, the appropriateness of the content and opinions regarding the intervention (e.g., “In your opinion, how did joining this therapeutic group helped to cope better with the loss of your loved one?”; “In your opinion, was there a turning point that allowed you to say that, in relation to the bereavement process, you have now managed to have more control over your emotions and manage them more adequately?”).

### 2.5. Data Analysis

Quantitative data were entered into SPSS Statistics software (version 28.0) and analyzed using descriptive and inferential statistical procedures, with pre- and post-intervention mean scores compared across the administered measures. The Reliable Change Index (RCI; Jacobson & Truax, 1991), using a significance criterion of 1.96, was calculated for the GMRI to assess the clinical significance of observed changes. Results from the remaining instruments were interpreted according to the cut-off scores established for the Portuguese population. The content of the group interview (fully transcribed) and the responses to the individual forms were analyzed through thematic analysis (Braun & Clarke, 2006) to identify the main strengths and aspects to be improved in the intervention from the participants’ perspective. All authors were involved in the analytic process, which included reviewing the responses, identifying preliminary codes and themes, and discussing and refining the thematic structure. Themes and interpretations were discussed collectively among the research team until consensus was reached.

## 3. Results

### 3.1. Quantitative Findings

Table 3 presents the pre-test (T1) and post-test (T2) GMRI results for the four patients. With respect to continuing bonds, clinically significant pre- to post-intervention changes were observed in Patient 1, indicating greater continuity in maintaining a meaningful bond with the deceased. Regarding personal growth, changes were identified in Patient 3, reflecting an increased perception of personal growth in the context of bereavement. Concerning feelings of emptiness and meaninglessness, significant differences between the two assessment points were found in three participants (Patients 1, 2 and 3), suggesting that after the intervention they were better able to attribute meaning to the loss and experienced reduced feelings of emptiness.

Regarding symptoms of anxiety and depression (Table 4), analysis based on the HADS cut-off scores indicated that, at the pre-test, two participants (Patients 1 and 3) presented moderate levels of anxiety and depression, while the remaining two participants (Patients 2 and 4) exhibited severe symptomatology. At the post-test, clinical changes were only observed in Patient 4, who demonstrated a complete absence of anxiety and depressive symptoms. Symptom levels in the other participants remained stable.

Regarding the presence of a possible diagnosis of Prolonged Grief Disorder (Table 5), outcomes varied across participants. According to the criteria for assessing the presence of the disorder, Patient 1 met the threshold for a possible diagnosis at pre-test. This classification remained at post-test; however, symptomatic improvement was observed, particularly with respect to intense emotional pain, episodes of anguish, and feelings of dazedness, confusion, and shock. At pre-test, Patient 2 also met the criteria for a possible diagnosis of Prolonged Grief Disorder. At post-test, no clinical improvement was observed, and symptom severity remained unchanged. Patient 3 did not meet the criteria for a possible diagnosis at pre-test but presented separation anxiety persisting for six months; this symptomatology was maintained at post-test. Similarly, Patient 4 did not meet sufficient criteria for a diagnosis of Prolonged Grief Disorder at pre-test, and post-test results indicated an absence of symptoms associated with the disorder.

### 3.2. Qualitative Findings

The thematic analysis of the responses to the individual forms and group interview revealed a higher number of strengths of the intervention identified by patients than the number of areas for improvement (Table 6).

#### 3.2.1. Strengths of the Intervention

One of the key strengths of the intervention identified by participants was its capacity to facilitate adaptation to loss and bereavement. Specifically, the intervention supported the integration of the loss into participants’ life narratives while maintaining a balanced level of emotional activation. It also promoted meaning-making in relation to loss-related emotions and enabled participants to experience the loss without pervasive feelings of guilt. Some participants also reported that the intervention helped them relate to memories of the deceased in a less distressing way, allowing them to recall their loved ones with greater acceptance and emotional comfort.

Evidence of adaptation to bereavement was observed when participants demonstrated acceptance of the reality of the loss, particularly during the expressive writing tasks involving letters addressed to the deceased. Through expressive writing, participants became more aware of the irreversibility of death and exhibited a range of emotional responses, progressing from more intense expressions of sadness toward greater acceptance and emotional relief.

“Being here has made us stronger to face our daily lives little by little, it has strengthened us.”

(Patient 2, Group Interview)

“It was a plus to be here, because it helped me to remember my grandmother with nostalgia, and not so much with the suffering that surrounded me.”

(Patient 4, Group Interview)

During the therapeutic process, it was noticeable that, as the patients were able to accept the loss and attribute meaning to it, they also developed an understanding of the universality of the psychological experience of loss. This process was accompanied by experiential learning about grief, facilitated through the shared reflections and experiences of group members.

“Sharing our pain and that of others has helped us to understand which stages of mourning we have already gone through and which we are still going through.”

(Patient 4, Individual Form)

“It helped a lot because there was dialogue between everyone, we were able to share our experiences and see that it’s not just us who are suffering and that there are other people suffering too.”

(Patient 3, Group Interview)

Through the activities carried out both during therapy and at home, we sought to increase patients’ awareness of a range of strategies available to them for managing painful emotions, in the present and future. Subsequently, patients began to report that they had employed particular strategies in moments of vulnerability, and that it had been very useful to them.

“We’re left with some tools that we’ll be able to use in the future. I even went back to the letter, I wrote a letter to my son again, it felt so good.”

(Patient 1, Individual Form)

“The tools we were given allowed me to evolve.”

(Patient 3, Individual Form)

One of the most frequently cited strengths by the patients was the opportunity to share. They recognized the possibility of sharing their experiences, emotions, and pain as a significant factor in alleviating grief-related symptoms and facilitating the progression of the grieving process.

“The sharing of pain, the sharing of emotions, knowing that we are not alone was very important.”

(Patient 1, Group Interview)

“The added value of this group turned out to be the sharing, which was very enriching and helped me to make irreversible and structured progress.”

(Patient 4, Individual Form)

In addition to sharing, the possibility of safe catharsis was identified by patients as another positive aspect of the intervention. It was also noticeable, and also something mentioned by patients, that both sharing and catharsis were facilitated by the small group size, which enabled the rapid development of a relationship among them.

“I felt safer and more secure to carry on.”

(Patient 2, Individual Form)

Another aspect highlighted by the patients, which was central to the success they reported, was the empathy, active listening and understanding they felt in the therapeutic context. As the relationship of trust was formed, the patients’ sharing involved more emotions and pain, as they felt protected and understood by the others. These dimensions were the pillars of the strengths highlighted by the patients, as they allowed for a deep involvement with the therapy, enabling improvements in the grieving process.

“I felt listened to and understood above all else, and the fact that they reassured me that I could cry allowed me to be more myself.”

(Patient 2, Group Interview)

“I felt that you helped me with that, I felt that you really gave the right word at the right time, which made me realize that you really listened to me, it did me good.”

(Patient 3, Group Interview)

As a result of the empathy and understanding experienced within the group, patients reported feeling sufficiently secure to both offer and receive support from one another. From a certain point in the intervention, it became evident that patients began to provide suggestions to assist one another, and even attempted to offer reassurance and comfort while others were sharing their pain.

“But it still allowed me to be available to try to make others feel less intense pain.”

(Patient 1, Group Interview)

“I also felt that, as I had been through the loss for longer, I was able to help in a different way, as I had already gone through certain stages of mourning, which my colleagues hadn’t.”

(Patient 4, Individual Form)

The heterogeneity of the group in terms of time since the loss provided patients who had experienced their loss more recently with an opportunity to cultivate hope and to envision a future beyond their grief. The possibility of projecting themselves into the future was experienced as a sense of relief, with patients recognizing that their feelings of guilt could be transformed without dishonoring the memory of the deceased.

“The fact that I’m in a group with people who have been through their loss for longer, and see the stage of grief they’re already at, gives me hope that one day it will be me, and that no matter how painful it is to live without the person we’ve lost, there’s always light in the coming months and years.”

(Patient 2, Group Interview)

#### 3.2.2. Areas for Improvement in the Intervention

The aspects identified by participants as requiring improvement in future interventions primarily concerned more regular feedback, adjustments to the intervention duration and greater group homogeneity. Regarding more regular feedback, several patients indicated that it would have been beneficial for the therapeutic letter provided at the end of the intervention to have been shared earlier and revisited during the treatment. This recommendation reflected a need for validation of their progress, as patients sought reassurance that, although negative emotions persisted, they were nonetheless evolving within their grieving process.

“I think that since you gave us the letter almost at the end, it might even be a good idea to write another letter in the middle of the therapy, letters could be delivered every two weeks, because the letter gave me a feeling of warmth.”

(Patient 2, Group Interview)

The length of the intervention was also discussed, but the patients’ perspectives on it differed. Most of the patients reported that the number of sessions was sufficient, but the patient who experienced the loss less recently said: “I think it could be longer, it could last a few more sessions.” (Patient 2, Individual Form). This patient expressed that with a few additional sessions, she could feel more confident about her progress and to better consolidate all of the content covered in the intervention.

Furthermore, participants suggested forming a more homogeneous group in terms of the time since the loss: “For me, I would have preferred to be in a group where the other members had suffered loss at a similar time to me.” (Patient 2, Group Interview). While a heterogeneous group may foster hope and future projection, it may also represent a factor that warrants careful consideration within the context of the grieving process.

## 4. Discussion

Cognitive-narrative intervention facilitates the integration of loss into the individual’s life narrative, supporting the construction of meaning related to both the loss and life itself (Lordello & Silva, 2021; Rocha & Gabriel, 2021). This process is achieved through the exploration and analysis of emotions, thoughts, and meaning-laden metaphors. The present study, with an exploratory and developmental nature, has allowed us to reflect on whether this type of intervention is feasible when dealing with grief related to deaths that occurred during the COVID-19 pandemic, considering the therapeutic group approach proposed here. In addition to developing the intervention, the study aimed to examine its procedural aspects by identifying strengths and areas for improvement, as well as to explore changes related to anxious and depressive symptoms associated with the grieving process and prolonged mourning, and to support the development of a system of meanings for the loss.

The findings showed that three of the four patients experienced a clinically significant reduction in levels of emptiness and meaninglessness after taking part in the intervention, indicating a better adaptation to bereavement. When an individual loses a loved one, there is a sense of incongruity between the loss and the perception of the world as fair and safe, which disrupts the system of meanings associated with the sense of peace (Milman et al., 2017). However, in the present study, there were no clinically relevant changes in the sense of peace in any of the patients. A study carried out by Andrade et al. (2017), highlighted the greater effectiveness of cognitive-narrative intervention compared to other approaches. This intervention facilitated adaptation to bereavement in their sample by achieving an adequate level of emotional activation and promoting changes in the meanings attributed to the loss. Similarly, the study conducted by Barbosa et al. (2014) recognized the important role of this therapeutic approach in the construction and attribution of meaning to the feelings and emotions associated with loss, thereby allowing for more effective grief management (Boelen et al., 2007). The findings of the group intervention presented here, in particular the clinical reduction in levels of emptiness and meaninglessness following participation, are in line with the literature and with similar interventions, albeit delivered individually. These findings underscore that the attribution or reconstruction of meaning in relation to the loss plays a key role in facilitating the process of adapting to bereavement (Gillies et al., 2014; Verdery et al., 2020).

Adaptation to grief/loss, although it does not change the reality of the loss, has important implications for transforming the quality of the grieving experience (Supiano & Luptak, 2014). Along with assigning and/or constructing meaning to the loss, the maintenance of an ongoing bond with the deceased is interpreted as an important aspect of successful adaptation to bereavement (Field et al., 2003), helping the bereaved to preserve a meaningful connection with the past (Fraley & Shaver, 1999).

According to Milman et al. (2017), the conflict with the sense of peace and continuous ties can jeopardize the adjustment to bereavement, triggering symptoms of prolonged mourning. In this sense, according to Field et al. (2003), it is important to consider that establishing a continuous bond with the deceased can lead to both adaptive and maladaptive variants. In the present study, only one participant demonstrated clinically significant improvement in terms of continuous attachment, although adaptation to bereavement or loss emerged as a notable strength reported by all the participants. The authors note that maintaining the deceased’s possessions intact since the time of death may reflect a maladaptive form of continuous attachment, and can indicate an incomplete recognition of the separation (Field et al., 2003). This behavior was observed in most of the patients who participated in the present study. Field et al. (1999) further describe that this form of continuous attachment exacerbates the symptoms of grief, i.e., the specific symptoms of bereavement and mood symptoms. In turn, deriving comfort from memories of the deceased—an experience reported by Patient 1 in the present study—represents an adaptive expression of continuous attachment, as it indicates greater recognition and acceptance of the separation (Field et al., 1999).

Regarding the symptoms of prolonged grief, only one patient showed improvements between the two assessment moments. Similar results were found for symptoms of anxiety and depression, which contrasts with findings reported in the literature on cognitive-narrative interventions (Rocha & Gabriel, 2021). The therapeutic power of elaborating metaphors and assigning meaning to loss—processes also present in the pilot intervention examined in this study—facilitates the expression and construction of emotions related to bereavement, which are essential for reducing grief-related symptoms (Rocha & Gabriel, 2021). Thus, although the quantitative results do not clearly or clinically indicate a reduction in anxiety, depression or prolonged grief symptoms in three of the four patients, qualitative analyses suggest improvement in this direction. It is therefore plausible that symptom reduction may only become evident at a later stage, rather than immediately at the end of the intervention, as assessed in the post-test.

A context lacking empathy may hinder the bereaved from resolving their grief and adapting to their loss (Paul, 1967). Conversely, in environments where reciprocal empathic expressions are common, feelings of relief and mutual understanding are more likely to emerge. Thus, a setting in which the individual feels listened to, supported, and understood encourages the expression of emotions related to the bereavement process (Paul, 1967)—a sentiment reported by participants in this intervention. Empathy, active listening and understanding were aspects that stood out in the qualitative evaluation of the intervention, as they enabled all participants to share in a way that was both enriching and safe, while also facilitating the collective construction of meaning (Rocha & Gabriel, 2021). It is well established that, after the loss of a loved one, individuals often feel confused and overwhelmed by the range of feelings and emotions associated with bereavement (Shear & Bloom, 2017). Therefore, when the bereaved seeks support to deal with their grief, they expect to find a context that is open to their pain, that can understand the process they are facing, and in a way, a context that can convey a sense of comfort (Shear & Bloom, 2017).

The opportunity to recognize the universality of the psychological experience of bereavement was also considered one of the key strengths of the intervention examined in this study, aligning with the advantages of group-based therapies described in the literature (Supiano & Luptak, 2014). In fact, engaging in a shared bereavement experience allows patients to learn that bereavement is a universal life event (Shear & Bloom, 2017). This sense of universality highlights the common elements present across individuals who experience a significant loss, namely longing, sadness, memories, and recurrent thoughts about the deceased (Shear & Bloom, 2017).

Recognizing the universality of the psychological experience of bereavement contributes to a change in the quality of grief, allowing for an attenuation of the intensity of bereavement-related emotions and feelings (Shear & Bloom, 2017; Supiano & Luptak, 2014). This recognition thus represents a significant therapeutic advantage, as acceptance of the meaning and consequences of the loss enables individuals to reconstruct their life narratives, adapt to the new reality and pursue future life plans in the absence of the deceased (Shear & Bloom, 2017).

The opportunity for sharing provided by the group intervention, as reflected in the results of this study, is also widely documented in the scientific literature. Sharing fosters the recognition of each individual’s narratives as similar to one another (Henoch et al., 2016), giving individuals the chance to share common experiences and feelings without judgment and to receive reinforcement for their efforts to cope with loss (Piper et al., 2009). This is particularly relevant given that these participants were grieving under conditions of restricted social contact and disrupted funeral practices due to the COVID-19 pandemic. Recognizing that others experience similar feelings may help individuals perceive their own emotions as less overwhelming and more manageable (Henoch et al., 2016), and convey a sense of safety in self-disclosure. Extending this perspective, Piper et al. (2009) emphasize the potential for personal growth and flourishing following loss that can emerge through shared experiences. Consistent with this view, Hogan et al. (1996) reported that bereaved individuals often describe personal growth during the bereavement trajectory in terms of a more meaningful evaluation of their lives, as well as an improvement in relationships with family members and close friends.

In fact, the literature on post-traumatic growth in the context of bereavement provides evidence that positive personal growth can occur following loss (Michael & Cooper, 2013), particularly in terms of a greater appreciation of life (Milman et al., 2017). Individuals who report greater life appreciation associated with personal growth also tend to exhibit changes in self-perception (Wagner et al., 2007), changes in interpersonal relationships (Gai et al., 2024), discovery of new life philosophies (Roberts et al., 2016), and attribution of a positive meaning to their identity and life (Milman et al., 2017).

This growth-related increase in valuing one’s own life is associated with improved functioning compared to the period prior to adaptation to bereavement (Gai et al., 2024). However, such considerations are not fully consistent with the results of the present study, as three of the four patients did not exhibit clinically significant changes in personal growth or in life valuation between the two assessment points. Only one patient reported clinically significant improvements in personal growth, which may be attributed to increased investment in closeness with other significant relationships and a shift in life perspective. Nevertheless, it should be noted that post-traumatic growth takes time to develop, and its emergence may not be evident within a short observation period. Consequently, the absence of more changes in the present study may reflect insufficient time for such processes to unfold. The inclusion of a follow-up (for example, 12 months post-intervention) could potentially gauge results at this level.

Regarding aspects of the intervention that could be improved, a discrepancy was found regarding group composition, particularly with respect to the time passed since the loss of the loved one. According to Piper et al. (2007), group composition plays a crucial role in therapeutic success and results from the articulation of individual characteristics. Groups in which members share the same primary characteristic are described as homogeneous, whereas those in which members differ in their primary characteristics are considered heterogeneous. Importantly, primary characteristics may vary across different levels of homogeneity (e.g., age, gender, and time since loss), which can promote opportunities for social learning within the group (Adler, 1995).

Although this type of homogeneity may promote change by enabling transference between group members, allowing them to project into the future, it may also generate anxiety (Adler, 1995) and evoke feelings of isolation from the rest of the group (Piper et al., 2007). In the present study, a more heterogeneous configuration of primary characteristics was adopted, as it facilitated the optimization of conflict areas and coping patterns (Adler, 1995), always focusing on the degree of vulnerability of each patient.

More regular feedback was also indicated by the participants as a point for improvement in a future intervention. In fact, feedback is included in the common learning factors for all psychotherapies, which is important for patients’ motivation levels (Lambert & Vermeersch, 2002). In the therapeutic context, the establishment of regular feedback is essential, as it contributes to the development of a sense of trust between the therapist and the patient, by ensuring that the therapist remains attentive to the patient’s progress and by highlighting improvements of which the patient may not be aware. As this sense of trust matures, anxiety related to uncertainties about the therapeutic process may be reduced (Lambert & Vermeersch, 2002). Future interventions could therefore benefit from incorporating structured feedback at key moments of the therapeutic process.

Participants’ views also differed regarding the duration of the intervention. Only one patient, who had experienced the most recent loss, indicated that a greater number of sessions would be beneficial. Nevertheless, some authors emphasize the idea that the time-limited nature of interventions reminds patients of the purpose of interpersonal relationships and therefore of life (e.g., Toth, 1997), thereby underscoring the benefit of brief interventions, especially in the context of bereavement. Specifically, Rocha and Gabriel (2021) argue that the brevity and structure of cognitive-narrative interventions foster therapeutic adherence. Although brief therapies are becoming increasingly common in the treatment of various psychological problems, evidence regarding their long-term benefits remains inconclusive (Piper et al., 2009).

### Limitations and Future Research

The main limitation of the present study concerns the small number of patients who participated in the intervention (*n* = 4) and the absence of a control group. However, this work was designed primarily as a pilot study focused on the development, implementation, and pilot-testing of an intervention. A larger sample size and more robust study design (e.g., including a formal sample size calculation and a control group) would be required to rigorously evaluate intervention effectiveness. Second, all the participants were being monitored in the Psychiatry department of the hospital where the intervention took place, which includes the Psychiatry and Psychology teams. It is hypothesized that the evaluation context may have interfered with the quantitative findings reported, insofar as the patients may have felt that reporting significant improvements in symptomatology could mean a break in follow-up in the Psychiatry service, even though the informed consent explained that participation in the study (or withdrawal) would not have any implications for clinical follow-up. Another limitation lies in the fact that the intervention facilitator/therapist herself carried out the final group evaluation interview, and her presence may have biased the evaluation of the intervention in the therapeutic group. Finally, all four participants were drawn from the same hospital psychiatry service, which is an important contextual factor that limits the generalizability of findings to community or primary care bereavement populations. In addition to further diversifying the target population, future studies should also consider a 6 or 12-month follow-up to assess the intervention’s potential long-term impact.

## 5. Conclusions

Although many bereaved individuals can integrate loss into their life narratives, a substantial proportion struggle to make sense of their experience. The COVID-19 pandemic, with its disorganizing character, has contributed to an increase in predominantly negative emotions in individuals and has hindered individuals’ ability to mobilize resources to cope with adversity. The cognitive and emotional components of the proposed intervention model gave patients enhanced participants’ awareness of the emotions that were triggered by the history of their loss. Although symptom reduction was not clinically significant for three of four patients, participants reported a sense of relief derived from attributing meaning to the loss, conveying an opportunity for psychological growth. The use of metaphors for their suffering and reconstructing personal narratives enabled patients to process the story of their loss. Finally, the opportunity for shared experiences within the group fostered a sense of mutual support among patients, a particularly important factor in bereavement contexts.

Despite the small sample size, by examining both quantitative findings and participants’ qualitative experiences of the intervention process, this study provides an initial contribution to the limited literature on group interventions for pandemic-related bereavement. Overall, this intervention format demonstrated promising potential for individuals whose grieving processes were shaped by pandemic-related constraints, which should be further explored in future studies involving larger and more diverse samples of bereaved individuals.

## Figures and Tables

**Table 1:** General structure of the intervention.

Session	Objectives	Activities	Between-Session Assignment
1	Introduce participants and establish group rules. Initiate recollection of the loss experience (Remembrance).	Presentation of participants and intervention goals. Establishing group rules (confidentiality). Icebreaker activity (“Who am I?”, “Who did I lose?”). Narrative exercise: telling the story of the loss.	“The story of my loss”: participants write about the thoughts and emotions experienced during the grieving process.
2	Explore the life narrative and process the story of the loss. Increase awareness of emotional and cognitive components of grief (Narrative reconstruction).	Sharing homework assignments. “Chapters of our lives” autobiographical exercise. Narrative reconstruction of the loss story. Exploration of emotions, bodily sensations, and associated thoughts.	Reflection on personal experiences related to the loss and identification of available support resources.
3	Promote meaning-making through metaphor and symbolic representation (Metaphorization).	Sharing homework assignments. Presentation of a meaningful object or photo related to the deceased. Metaphorization exercises (“Title of the book”, “My house after the loss”). Letter writing to the deceased (“Hello again”).	Write a response letter from the perspective of the deceased.
4	Reconnect emotionally with the deceased and address unresolved issues (Resonance).	Sharing homework assignments. Imaginary conversation with the deceased. Exploration of emotional memories and unresolved feelings.	Reflection on personal progress and emotional changes during the intervention.
5	Identify personal and community resources and reconstruct future narratives (Projection).	Sharing homework assignments. Therapist’s letter describing participant’s progress. Narrative projection exercises (“What now?”).	Prepare reflections on personal changes experienced during the intervention.
6	Review personal and community resources and reconstruct future narratives.	Reflection on participant’s journey using evocative images. Reading of therapist letters.	

**Table 2:** Participants’ sociodemographic data and data related to the situation of loss.

Participant	Gender	Age (Years)	Civil Status	Time Since Death	Characteristics of Loss
Patient 1	Female	59	Married	9 months–6 months	Bereavement due to the sudden death of her son at the age of 33. Vulnerability factors included restriction of the funeral ritual, inability to attribute meaning to the loss, feelings of anger and voluntary isolation.
Patient 2	Female	55	Widowed	5 months–3 months	Bereavement due to the sudden death of her spouse at the age of 57. Vulnerability factors included an inability to attribute meaning to the loss, feelings of guilt and anger, and reduced social support.
Patient 3	Female	65	Divorced	1 year or more	Mourning the natural and early death of her mother, due to a prolonged illness. Vulnerability factors included inability to attribute meaning to the loss, reduced social support, restriction of the funeral ritual and voluntary isolation.
Patient 4	Male	18	Single	1 year or more	Mourning the death of her grandmother naturally and suddenly. Vulnerability factors included inability to attribute meaning to the loss, restriction of the funeral ritual and voluntary isolation.

**Table 3:** Differences between the Pre (T1) and Post-test (T2) in the GMRI.

GMRI Subscales	Participant	T1	T2	RCI ^1^
Continuing bonds	Patient 1	9	4	**2.06**
	Patient 2	7	7	0
	Patient 3	7	8	0.41
	Patient 4	1	4	1.23
Personal growth	Patient 1	3	9	1.79
	Patient 2	8	1	1.34
	Patient 3	0	5	**2.23**
	Patient 4	4	8	1.79
Sense of peace	Patient 1	6	7	0.43
	Patient 2	5	5	0
	Patient 3	5	6	0.43
	Patient 4	6	7	0.43
Emptiness and meaninglessness	Patient 1	5	3	**2.85**
	Patient 2	2	8	**5.69**
	Patient 3	4	4	**3.56**
	Patient 4	3	5	0.71
Valuing life	Patient 1	6	5	−0.47
	Patient 2	5	6	0.47
	Patient 3	4	4	0
	Patient 4	2	3	0.47

^1^ Significant clinical differences (*p* < 0.05) are marked in bold.

**Table 4:** Differences between the Pre (T1) and Post-test (T2) in the HADS.

HADS Subscales	Participant	T1	T2 ^1^
Anxiety	Patient 1	12	11
	Patient 2	18	19
	Patient 3	12	14
	Patient 4	19	**2**
Depression	Patient 1	15	16
	Patient 2	19	19
	Patient 3	14	14
	Patient 4	11	**1**

^1^ Improvements in symptoms are marked in bold.

**Table 5:** Differences between the Pre (T1) and Post-test (T2) in the PG-13.

PG-13 Subscales	Participant	T1	T2 ^1^
Separation anxiety	Patient 1	Several times a day	Several times a day
	Patient 2	Several times a day	Several times a day
	Patient 3	Several times a day	Several times a day
	Patient 4	At least once a day	**Almost never**
Cognitive, emotional and behavioral symptoms	Patient 1	Several times a day	**At least once a week**
	Patient 2	Daily intensity	Daily intensity
	Patient 3	Fairly	Fairly
	Patient 4	At least once a day	**Slightly**
Social and occupational dysfunction	Patient 1	Yes	Yes
	Patient 2	Yes	Yes
	Patient 3	No	No
	Patient 4	No	No

^1^ Improvements in symptoms are marked in bold.

**Table 6:** Themes and sub-themes identified through thematic analysis and number of participants who mentioned each sub-theme.

Themes	Sub-Themes	*n*
Strengths of the intervention	Coping with grief/loss	4
	Acquiring therapeutic tools	4
	Sharing and safe catharsis	4
	Empathy, active listening and understanding	4
	Diverse group composition in relation to loss	4
	The universality of psychological experience	3
Areas for improvement	Regular feedback	1
	Duration of the intervention	1
	Homogeneous group composition in terms of loss	1

## Data Availability

The data are available upon request to the corresponding author.
